# Supplementary material for: Validation of the Spanish Version of the Fear of COVID-19 Scale (FCV-19S) in Long-Term Care Settings
Source: Int J Environ Res Public Health. 2022 Dec 3;19(23):16183. doi: 10.3390/ijerph192316183 (PMC9741095; doi:10.3390/ijerph192316183)
Supplement: Supplementary file 1 [file ijerph-19-16183-s001.zip › ijerph-2024617-SI.pdf]

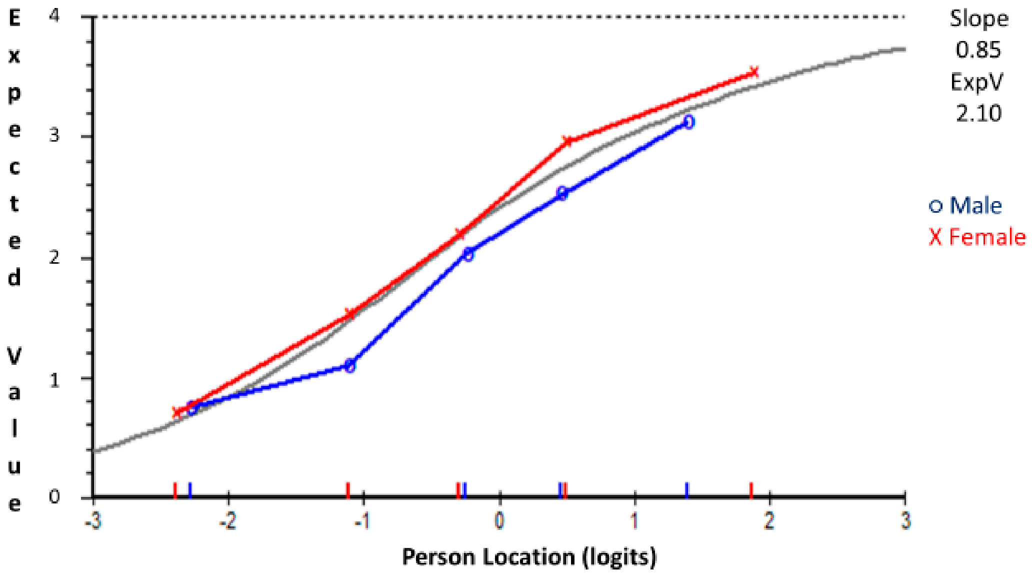

Figure S1. Differential item functioning for item 5, by sex. Note: The person locations along the construct continuum, expressed in logits, in showed for both males and females. For the same construct level, males display significantly lower scores than females.

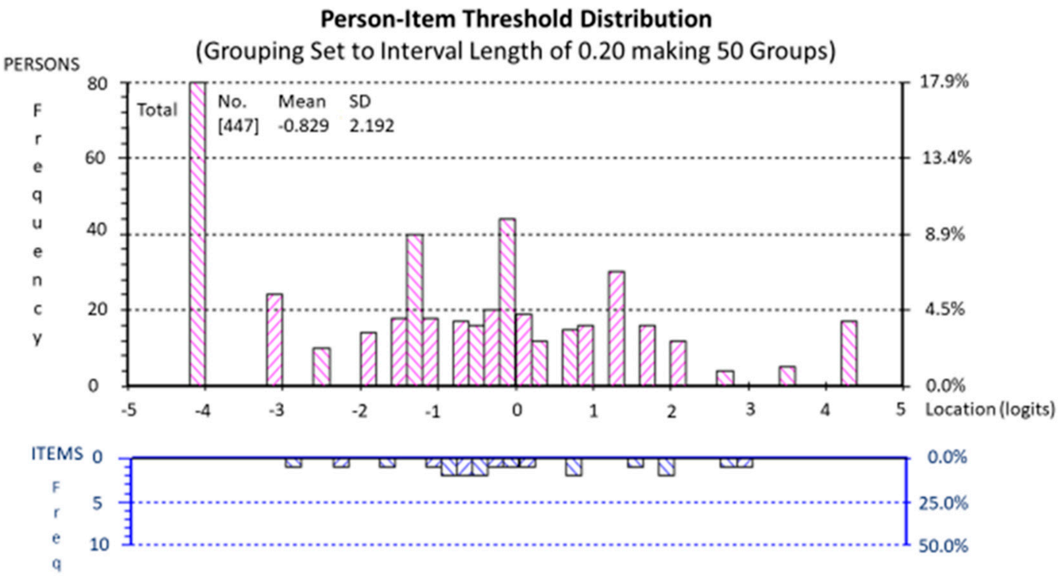

Figure S2. Person-item threshold distribution of the FCV-19S. Note: the distribution of persons (top) and item (bottom) threshold locations are shown on the same logit scale. Persons with higher fear level and more difficult item thresholds are represented on the right.
